# Comparison of frictional resistance between passive self-ligating brackets and slide-type low-friction ligature brackets during the alignment and leveling stage

**DOI:** 10.4317/jced.55913

**Published:** 2019-07-01

**Authors:** Sandra-Liliana Gómez-Gómez, Junes-Abdul Villarraga-Ossa, Juan-Gustavo Diosa-Peña, Juan-Fernando Ortiz-Restrepo, Robinson-Andrés Castrillón-Marín, Carlos M. Ardila

**Affiliations:** 1 Orthodontics; Master in Epidemiology; Assistant Professor, School of Dentistry, Universidad de Antioquia; 2 Mechanic Engineer. Professor, Universidad de Antioquia; 3 Orthodontics, Universidad de Antioquia; 4 Orthodontics. Professor School of Dentistry, Universidad de Antioquia; 5 Ph.D in Epidemiology; Biomedical Stomatology Group, Universidad de Antioquia, Medellín, Colombia

## Abstract

**Background:**

To compare the frictional resistance between passive self-ligating brackets and conventional brackets with low-friction ligature under bracket/archwire and root/bone interface during dental alignment and leveling.

**Material and Methods:**

A tridimensional model of the maxilla and teeth of a patient treated with conventional brackets, and slide ligatures was generated employing the SolidWorks modeling software. SmartClip self-ligating brackets and Logic Line conventional brackets were assembled with slide low-friction ligatures, utilizing archwires with different diameters and alloys used for the alignment and leveling stage. Friction caused during the bracket/archwire interface and stress during the bone/root interface were compared through a finite element model.

**Results:**

SmartClip and Logic Line brackets with slide elastomeric low-friction elastomeric ligature showed similar frictional stress values of 0.50 MPa and 0.64 MPa, respectively. Passive self-ligating brackets transmitted a lower load along the periodontal ligament, compared to conventional brackets with a low-friction ligature.

**Conclusions:**

Slide low-friction elastomeric ligatures showed frictional forces during the bracket/archwire interface similar to those of the SmartClip brackets, while the distribution of stresses and deformations during the root/bone interface were lower in the passive self-ligating brackets.

** Key words:**Orthodontic friction, finite element analysis, orthodontic brackets, orthodontic wires.

## Introduction

Frictional resistance is the force that delays or impedes movement between two objects that are in contact (1). In orthodontic biomechanics, the capacity of the archwire to slide through brackets and tubes is essential to achieve proper alignment and leveling since dental movement is only possible when the stress applied exceeds the friction caused during the bracket/archwire interface (2). High levels of frictional force between the slot and the archwire may cause binding of these two components, which would result in a shallow (if any) dental movement. Slight forces are deemed as optimum during orthodontic treatment because they maintain anchorage, allow more significant dental movement, and decrease radicular reabsorption risks (3-5).

Friction emerging during an orthodontic treatment is multifactorial and can be caused by physical factors, such as the type of bracket (6), the size and alignment of archwire (7), the ligating method (8), and other biological factors, such as the saliva, bacterial plaque, and food remains, among others (9-10).

Several methods have been proposed to decrease the friction resulting from the ligation of archwire against brackets, including self-ligating brackets and nonconventional ligation systems. Self-ligating brackets have been designed with select accessories like clips or lock gates, which keep the archwire in the slot, unlike conventional ligation, which is conducted with elastomeric or metallic ligatures.

The advantages of self-ligating systems that have been reported are: the patient comfort, simplifies oral hygiene, less clinical care, and shorter treatment time (11). However, there are certain disadvantages, such as incapacity to express torque, frequent clip failures, larger size, and higher cost than conventional brackets (12). Nonconventional ligatures have been developed to improve some deficiencies of the self-ligating brackets (such as Leone Slide low-friction ligatures), which can be adjusted to brackets like a conventional elastomeric ligature, but simulating the behavior of self-ligating systems, allowing a presumably free sliding of the archwire along with such ligatures (13).

The objective of this study was to compare the behavior, in terms of frictional resistance, during the bracket/archwire and bone/root interface, between passive self-ligating brackets, and conventional brackets with Slide-type low-friction ligatures during the alignment, and leveling stage.

## Material and Methods

The following methodological sequence was conducted: the setting of mechanical characteristics of orthodontic materials, clinical treatment, construction of computer-assisted design (CAD) model of biological structures, and orthodontic accessories, assembly of systems, movement simulation, results, and comparison of the results.

-Initial Clinical Treatment

A patient with no systemic commitment was chosen; proper oral-dental conditions and slight dental crowding were mandatory; then, the orthodontic treatment was applied through the assembly of the following accessories: a Logic Line (Leone® Florence, Italy) MBT bracket system, slot 0.022” x 0.028” with Leone® SlideTM low-friction ligatures; a sequence of Ni-Ti 0.016”, 0.017”x0.025, 0.019”x0.225 archwires, β-Ti 0.017”x0.025”, and stainless steel 0.019”x0.025”. A conic beam CT of the patient was taken immediately after the assembly of orthodontic appliances (T1) and upon completion of the alignment and leveling stage (T2), which served as the basis for the digital modeling.

-Mechanical Characteristics of Orthodontic Accessories

Physical tests were conducted to determine the tension/deformation mechanical characteristics of the alloys employed (stainless steel, β-Ti, Ni-Ti) with an Instrom AN8032 universal machine (Analógica Instrumentação e Controle Ltda., Belo Horizonte, MG, Brazil); a tension force was applied. The load cell has a maximum capacity of 222.4N, and the machine was operated at 0.075mm/sec.

Five assays were conducted on each alloy (stainless steel, β-Titanium, Nickel-Titanium), and an archwire (0.016”, 0.017x0.025”, 0.019”x0.025”) with a total of 35 assays. The information obtained was entered into the ANSYS 14® (Pittsburgh, USA) simulation software.

-Model of Maxilla/Teeth System 

The image of the maxilla/teeth system was obtained through a cone beam computed tomography (CBCT) taken of the patient, before starting the alignment, and leveling (T1) stage. The CBCTs were taken with a SIRONA XG5-3D scanner (SIEMENS, Texas, USA) with a maximum window of 8x8 cm, high resolution, and 0.16 mm isotropic voxels; 2-5 sec exposure time, and 60-90 Kv and 3-16 mA transmitter. CBCTs provided a Digital Image and Medical Communication (DICOM) image file of the maxilla/teeth system with a 0.5 mm resolution per cut. From these images, the CAD 3D model of the scanned maxilla was built, processing the DICOM image with an Autodesk Fusion 360 (San Francisco, CA, USA) software, for the construction of a point cloud of the maxilla/teeth system from the CBCT obtained with the CAD scan.

Construction of Bracket, Archwire, and Ligature Models 

Models of the two types of brackets, stainless steel, NiTi, and β-Ti archwires from the company 3M UnitekTM (Monrovia, CA. USA) and Leone® (Florence, Italy) low-friction slide ligatures were built, employing the CAD module of SIEMENS NX (Plano, Texas, USA) from photographs taken with a NIKON SMZ 1000 (Kings, London, UK) stereo microscope. These photographs were used to support the reconstruction of the models of each element of the system, each of which was assembled according to the exact spatial distribution shown on the patient’s scan.

-Assembly of the maxilla/teeth-bracket/archwire/Ligatures System

**Figure 1:**
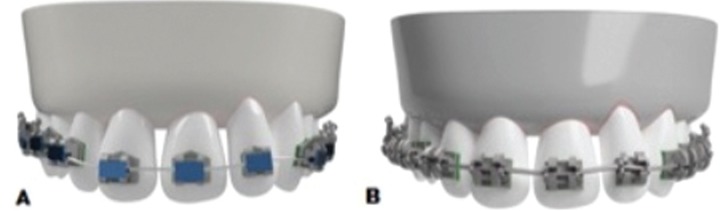
Assembly of the systems. A. Logic Line with Slide ligatures. B. SmartClip.

After obtaining the CAD models of the systems, the virtual assembly of the whole system was used with the 2010 Solid Works software for simulation, through the finite element model (FEM) (Fig. 1).

-FEM Simulation

After the assembly of the CAD models with the bracket/archwire/ligature systems of SmartClip and Logic Line, the simulation was used to obtain stresses and nodal displacements. The construction of the FEM required the meshing of the assembly previously obtained, using the CAD models to discretize the entire system into more straightforward elements. Further, border conditions (loads and supports) and mechanical properties of materials and biological structures were applied in Young’s modulus and Poisson’s modulus (14). Later, the calculation module provided in the FEM application was used for the resolution of the model, and for obtaining data on displacement, stresses, and loads. Stresses were used in the bracket/archwire interface as an indicator of the frictional resistance of these systems. Since the geometry of the slot and archwire was not modified within the simulation, regular and friction forces had a direct influence on the value of these stresses.

The study protocol was approved by the Institutional Review Board. Also, an informed consent was obtained from the patient.

## Results

-FEM Validation

To authentically represent the reality of the subject patient in the software, CAD models were assembled with the same alignment characteristics of the patient After completing the torque expression, and after three months of the adaptation of archwire SS 0.019x0.025”, the final CBCT was taken (T2). Reference planes were established with Galileos software (Dentsply Sirona Inc., PA, USA) in order to evaluate in millimeters the dental changes generated on the three spatial planes. The reference planes were: a strict horizontal on the frontal plane to evaluate vertical changes; the panoramic function of software 0° in a coronal view to evaluate rotational dental changes, and a vertical line from an axial view going along the apices of incisors roots in order to evaluate sagittal changes.

-Comparison of CBCT Movements versus Simulation

When CBCT in T2 was evaluated, it was noted that dental leveling was achieved, mainly due to extrusive movements of posterior segments and the relative intrusion of central incisors, being the first premolars the maximum point of vertical change on the CT. These results are comparable to the movements obtained during the simulation of both bracket systems with 0.017x0.025,” and 0.019x0.025” NiTi archwires; stresses and deformations were specifically found in apices and necks of premolars and first molar. The calculation of compression, in supporting structures, before the force applied was made through the assessment of FEM chromatic scales, which go from blue to red, being the blue areas those with lower stress and the red showing higher stress.

Additionally, when alignment in T2 was achieved, it was noted that the most significant rotational changes achieved were those in pieces 12 and 22 in an mesovestibular direction. These were the teeth that were showing the most malposition in the patient’s initial condition. These movements are also correlated with the findings of the friction seen during the bracket-archwire interface in the simulations, where the highest amount of frictional force in the entire system was generated, at the level of piece 12, which may result from the highest critical contact angle caused in such a tooth, since it was more rotated than its adjacent pieces.

These results confirm that the generated friction, and the stresses and deformations derived from the simulated systems, accurately represent the clinical condition of the patient.

-Mechanical Properties of Materials

**Figure 2:**
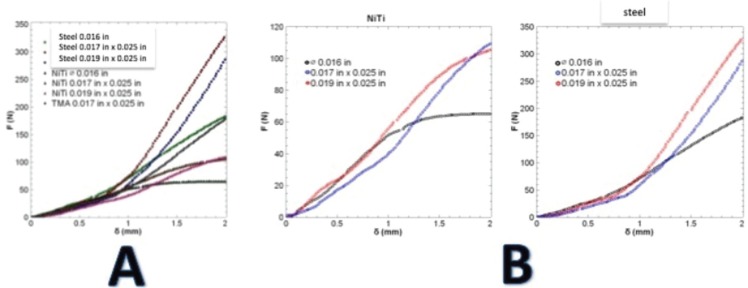
A. The rigidity of archwires at 2 mm traction. B. Tension curve of NiTi and steel archwires.

Results of the traction test of materials are shown in Figure 2A, which shows the values of the resistance to the traction of the tested archwires, subdivided according to their manufacture material, and the cross-section size. These mechanical tests showed a meaningful difference in the tension resistance among all tested archwires, 0.019x0.025” being the highest force generated by the stainless-steel alloy, with 448.644 N in the two mm displacement, while the lowest force was seen in the NiTi archwires (0.016”), with 68.894 N in the same displacement.

It was found that the stretching resistance of archwires with the same cross section increased as the rigidity of its construction material increased.

Archwires may be three times more rigid than NiTi archwires of the same shape and cross-section. Significant differences were also found in the traction resistance among the archwires made with the same material, but with different cross-section sizes, where the 0.016” stainless steel archwires exercised a force of 180N/mm, while the rectangular ones (0.019x0025”) exercised a force of 335N/mm before fracture. This is a behavior similar to that of the NiTi archwires with 63.7N/mm for the 0.016” diameter, and 105 N/mm for those with 0.019x0.025” (Fig. 2B). Data obtained from the traction tests of the materials were useful in performing the friction simulation between the two bracket systems evaluated.

-Frictional Evaluation

Friction Generated during the Bracket/Archwire/Ligature Interface

Frictional force generated by the Logic Line brackets with Slide-type low-friction ligatures and the entire sequence of archwires showed an average of 0.64Mpa. This frictional force was produced during the entire alignment and leveling stage with values similar to those of friction caused by SmartClip passive self-ligating brackets (0.50MPa).

When the types of material were compared in the descriptive analysis, Nickel-Titanium showed higher frictional force, on both bracket systems, while Titanium-Molybdenum archwires showed the lowest levels of resistance to sliding. When both orthodontic systems were compared with those alloys, NiTi 0.019”x0.025” archwires showed the highest frictional force rates, with the same behavior in SmartClip brackets and Logic Line brackets with Slide ligatures (1.34674 and 1.3467 MPa, respectively); on the other hand, the combination 0.016” steel archwires and SmartClip brackets with 0.126192 MPa, generated the lowest friction ( Table 1).

**Table 1:**
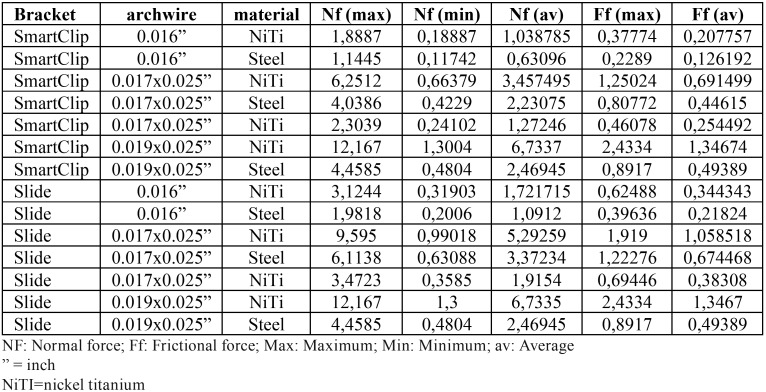
Descriptive statistics of the frictional stress generated in MPa in both bracket systems.

**Figure 3:**
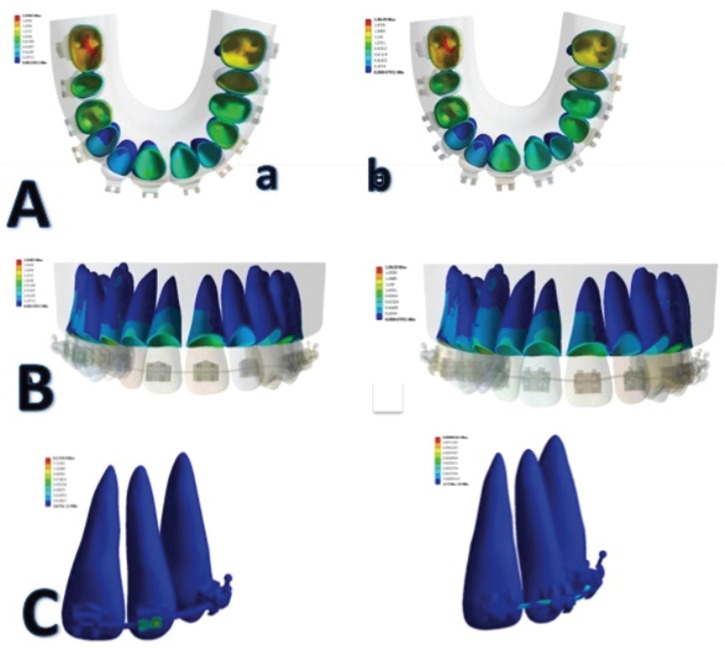
A. Distribution of stresses on the root by both brackets systems: a. Logic Line with Slide ligatures. b. SmartClip. B. Friction Generated during the Bone/Periodontal Ligament Interface. C. Single deformation during the bracket/archwire interface.

When unitary deformation was assessed with the quantification of the FEM chromatic guide, a higher distribution of stresses was observed during the bracket/archwire interface on lateral incisors, especially during extrusive movements of these teeth, during the leveling stage with 0.019x0.025” NiTi archwires (Fig. 3A).

The dental pieces that showed the lowest friction during the bracket/archwire interface were the first premolars in all arch combinations.

Friction Generated during the Bone/Periodontal Ligament Interface (Fig. 3B).

When the chromatic distribution guide of stresses and deformations transmitted by the periodontal ligament to the bone was evaluated, a higher load was observed on posterior teeth, primarily on the first molars, and secondarily on the second premolars. This behavior could be associated with a decreased leveling of these teeth, or to an increased proportion of the dental structure of such pieces, which could require a higher concentration of stresses on the periodontal ligament, then, the mobilization can be made during the orthodontic treatment.

Single deformation during the bracket/archwire interface 

When total deformation generated by both bracket systems was compared during the root/periodontal ligament interface, no significant differences were observed; however, passive self-ligating brackets seem to reduce the load transmitted through the periodontal ligament, especially in those teeth subject to vertical movements, such as first premolars or lateral incisors, which required rotation and extrusion movements to correct their malposition. Finally, Figure 3C shows how total deformation and single deformation had a higher concentration in the radicular cervical third on anterior teeth.

## Discussion

In this study, the results of the frictional force generated during the alignment and leveling stage were similar with 3M® SmartClip passive self-ligating brackets and conventional brackets, with Leone® Slide low-friction ligatures, in all combinations of archwires, and frictional forces of 0.50MPa and 0.64MPa, respectively. These findings are similar to those found in a previous study (5), where the same bracket/ligation systems showed the same frictional force behavior during the displacement of 0.019x0.025” steel archwires. One possible explanation for this physical behavior is that the SmartClip brackets were provided with a regular force in all the archwire configurations employed by the NiTi active clips. This probably caused frictional force levels, similar to those generated by Slide ligatures, which make conventional brackets resemble tubes, as was reported earlier (13).

The similarity of frictional force generated in both systems can also be explained by the fact that they have the same MBT bracket system prescription, which may be caused by the fact that factors such as angulation and torque did not modify their frictional behavior. In the same manner, both bracket systems show similar diameters; then, the distance between brackets did not make a significant difference in the friction resulting from both systems. However, Kumar *et al.* (15) found different results, where despite Slide ligatures, minimum levels of frictional force were shown, and the SmartClip brackets showed a lower sliding-resistance behavior. The difference in this research lies in the methodology employed, since authors conducted several assays with a universal Instrom traction machine in different types of bracket/archwire combinations, considering static and kinetic friction, and not factors such as critical contact angle or alloy properties. When linear sliding was performed, it was not considered that, when an archwire is in contact with two points of slot walls, and when the bracket is angled with respect to the archwire (as during the alignment and leveling stage), the deflection begins to contribute to sliding resistance. The angle resulting between the archwire and the slot is bigger than that known as critical angle (16), and a plastic deformation arises; this is added as a component of the frictional resistance. Another potential explanation could be given for the discrimination of the results offered by the FEM, as compared with the Instron machine, which only provides final reports, such as the summation of several events.

Disadvantages mentioned above were not presented in this study since the mathematical method employed in the FEM provides more accuracy than other researches in vitro methods (17). The accuracy and validation of the FEM has been demonstrated in previous studies (18); then, this method can be considered as a valid model to assess the frictional force in different bracket/archwire combinations.

An important result of this study is that, in addition to the ligation method, the alloy of archwires employed, and the size of the cross-section played a significant role in the levels of frictional force developed during the alignment and leveling stage. It was noted that, as the cross-section size increased, the friction generated increased proportionally.

This can occur because the contact area between surfaces increases the friction coefficient, and the reasonable force applied, which is compatible with the results found in several studies (19-21). Additionally, the Ni-Ti and Ti-Mo alloys showed friction levels higher than the stainless steel archwires, since resilience of such archwires is more likely to generate binding and notching; besides, the friction coefficient of these alloys is also higher in contact with stainless steel brackets (1-4,6). Another potential explanation for this result is the surface of the analyzed alloys, where structural irregularities of archwires affect the resulting friction coefficient.

Therefore, it has been shown that TMA and NiTi archwires can produce more friction than stainless steel (22); scanning electron microscopes have proven that they show several areas with holes, craters, and less high areas than stainless steel archwires, which produce a smooth and uniform surface.

Another objective of this study was to evaluate the distribution of stresses during the bone/root interface of both ligation methods. For this purpose, a periodontal ligament/non-linear bone system was used (23); this permits a closer approach to clinical reality. First, it was found that, for both bracket systems, the distribution of stresses showed a higher concentration on neck and root, especially on anterior teeth, which can be explained by the fact that this is the area of closer proximity to the of application of the stress; the middle area of periodontal ligament shows lower rigidity than the apical (2). Additionally, and bearing in mind the distribution of loads on the root surface during the uncontrolled inclination, where center of resistance and rotation coincide during the initial alignment stages, the uncontrolled inclination results in a distribution of higher load on the neck, where the expression of rotational tendency of this type of movement starts (24).

When the FEM chromatic scale was evaluated, comparing the distribution of stresses and deformations between SmartClip brackets and Slide ligatures, a higher concentration was observed in the models applied with non-conventional elastomeric ligatures. This seems to indicate that surrounding biological tissues respond more physiological to the stress applied by SmartClip brackets during dental movement. This can be compared with the in vivo study of Reddy *et al.* (25), where SmartClip brackets showed higher efficiency in terms of treatment time than Slide low-friction ligatures, which resulted in a lower alignment and leveling time.

Clinical implications of this research suggest that the Slide low-friction ligatures can be an alternative for passive self-ligating brackets, since they are more accessible and show similar physical behavior in terms of frictional force, and this could result in more physiological forces when compared to the conventional ligation, more frequently used in orthodontic practice.

Finally, the limitations of the finite element method should be taken into consideration, since biological factors affecting friction, such as saliva and bacterial plaque, among others (26-28), were not subject to analysis in this research, because the FEM is an entirely mathematical method which does not allow for the inclusion of such variables. In the same manner, the molecular or physical structure of Slige ligatures was not included in calculating the friction generated by them. The research only included the CAD design, which contains the structural figure, but not its actual oral behavior.

The study of Mendes *et al.* (29) about chemical and physical properties during the exposure of this type of ligatures to the oral environment conditions found that, as time passed, the friction generation decreased; the best option to reduce friction is the non-conventional ligatures (30), then, it is important to assume these factors for future researchers.

## Conclusions

SmartClip-type passive self-ligating brackets, and conventional brackets with Slide-type low-friction ligatures, showed similar frictional forces during the bracket/archwire interface, dental alignment and leveling, Regarding the distribution of stresses and deformations on bone and periodontal ligament, of both systems, the passive self-ligating brackets could provide more homogeneous distribution forces, which may be related to more physiological movements.
